# From ‘Little Things’ to ‘Seagulls’: A Mixed Methods Exploration of Patients' Experiences of Person‐Centred Compassionate Care in General Medical and Surgical Wards

**DOI:** 10.1111/hex.70844

**Published:** 2026-08-30

**Authors:** Ruth Cox, Jayne Hewitt, Emilia Dauway, Alex McConnell, Jodie Nixon, Faiza El‐Higzi, Sanjeev Naidu, Elizabeth Ward

**Affiliations:** ^1^ Queen Elizabeth II Jubilee Hospital Metro South Health Coopers Plains Queensland Australia; ^2^ Griffith University, School of Nursing and Midwifery Gold Coast Queensland Australia; ^3^ Consumer Partner Queen Elizabeth II Jubilee Hospital and Metro South Health Brisbane Queensland Australia; ^4^ Metro South Health Clinical Governance Risk and Legal Brisbane Queensland Australia; ^5^ The University of Queensland, School of Health and Rehabilitation Sciences Brisbane Queensland Australia; ^6^ Centre for Functioning and Health Research Metro South Health Buranda Queensland Australia

**Keywords:** compassionate care, consumer and community involvement, hospital, patient experience, person‐centred care

## Abstract

**Background:**

Person‐centred compassionate care (PCCC) shapes the quality of relationships between health care professionals (HCPs) and patients, influences how healthcare is experienced, and ultimately can impact patient outcomes and HCP satisfaction. There is limited evidence about patient perspectives of multidisciplinary PCCC in acute general medical and surgical wards.

**Objective:**

This study aimed to explore patients' perceptions of PCCC to inform co‐design of an intervention to enhance PCCC in general medical and surgical wards.

**Methods:**

Sixty (60) inpatients on a general medical or general surgical ward completed the Sinclair Compassion Questionnaire (SCQ) three times—considering their interactions with doctors, nurses, and allied health separately. They also completed five qualitative questions about PCCC. An additional 20 patients or their carers were interviewed exploring the same five topic areas. Reflexive thematic analysis was used to analyse qualitative data.

**Results:**

The median SCQ score was high (4.5; SEM = 0.05) indicating that participants perceived high levels of compassion from HCPs during their hospital admission. Fewer than 20 per cent (17.9%) of the SCQ scores were less than 4 out of 5 and more than half (51.2%) were 4.5 or higher. Analysis revealed no significant differences (*p* = 0.066) between professional groups (Doctors: median = 4.2, SEM = 0.10; Nurses: median = 4.75, SEM = 0.07; Allied Health: median = 4.25, SEM = 0.09). There were no significant differences in scores (*p* > 0.01) by ward type, length of stay, cultural diversity, or disability. Females rated allied health staff as significantly higher on SCQ (median = 4.9, SEM = 0.1) than males (median = 4, SEM = 0.15) (*p* = 0.003). Qualitative responses indicated many recommendations to enhance care. From ‘little things’ to ‘seagulls’ was a phrase co‐created by the research team to encapsulate the range of participant perspectives across the themes. Co‐produced themes and key messages to staff focussed on being in the moment, finding out about the whole patient and their journey, partnering with the patient to the level they choose, knowing that patients make allowances for the pressures on staff, and that small acts of kindness are critical to the patient experience.

**Conclusion:**

This co‐produced research privileged the voice of a diverse group of patients and provided new and actionable insights regarding PCCC across HCPs in acute hospital general medical and general surgical wards. Findings emphasised that SCQ scoring was a useful ‘temperature check’ regarding PCCC indicating that some improvements were needed. However, there may be a ceiling effect for the tool which requires further research. Qualitative data indicated where improvements were needed.

## 1 Introduction

Person‐centred care improves patients' healthcare experience [1, 2] and the efficiency and effectiveness of healthcare systems [3, 4]. As a multifaceted concept, person‐centred care encompasses an ethical imperative for care to be shaped by peoples' identities and social contexts beyond healthcare encounters [2, 5]. Respect for each patients' needs, values, and preferences, with recognition of patient and family capabilities, along with collaborative and respectful communication, are pivotal to person‐centred care [6, 7]. Compassion is also central to person‐centred care [6, 8]. It is defined as ‘a virtuous and intentional response to know a person, to discern their needs and ameliorate their suffering through relational understanding and action’ [9, p. 5]. Compassion enables health care professionals (HCPs) to provide the holistic care that is valued by patients, families, and HCPs [10]. It is fundamental to shaping nurses' professional quality of life [11]. Compassion is increasingly considered a quality indicator by patients, families, policy makers, healthcare organisations, and governments [12]. Conversely, where compassion is lacking, patient and family complaints increase, and healthcare costs are higher [13, 14]. Hence, it is recognised that how the concepts of person‐centredness and compassion are integrated into healthcare—from here on referred to as ‘person‐centred compassionate care’ (PCCC)—can shape the quality of relationships between HCPs and patients, influence how healthcare is experienced, and ultimately can impact patient outcomes [8, 14].

Meanings and enactment of PCCC can differ across healthcare settings [3, 4, 10]. Most people admitted to hospital are cared for on medical or surgical wards [15], and so attention on these settings is important. A recent scoping review focusing on empathic and compassionate healthcare interactions found that most studies occurred in primary care or palliative care [16]. Similarly, a systematic review exploring compassionate care in medicine identified only three studies conducted within hospital settings [14]. Of these, one study found that less than 50% of HCPs reported that they provided good compassionate care [17]. Another study found that patients scored a compassion questionnaire higher in acute care and hospice settings compared to long term care [12]. However, the type of acute care ward(s) was not specified. Conversely, there is more literature examining person‐centred care in hospital settings. A recent meta‐narrative review identified 124 articles which focussed on person‐centred care in hospitals [18]. The review highlighted that attributes and behaviours of HCPs, such as compassion, empathy, and effective communication were facilitators of person‐centred care, but these could be jeopardised by organisational factors such as workplace culture, systems, and policies [18]. The authors noted that there was a need to move from generically reviewing person‐centred care across hospitals to focusing on specialised care settings; multidisciplinary groups; patient and family perspectives; and to explore patient‐related factors that influence PCCC [18].

The issue that many studies conducted to date have not focussed on, is the patient perspective when examining PCCC [16, 18]. There are some limited examples, including an international online survey where participants described their experiences of compassionate encounters with HCP across hospitals, private consulting rooms, and hospices [19]. Themes highlighted the enduring impact of empathic care with verbal and non‐ verbal communication, HCP attitudes and attributes, and small, thoughtful gestures being important [19]. In another online study from New Zealand, participants reported that listening and paying attention, following‐up and running tests, continuity and holistic care, respecting preferences, genuine understanding, body language and empathy, and counselling and advocacy made them feel cared for by their physician [20]. The physicians in this study were almost exclusively in primary care. A recent scoping review on compassion in healthcare identified only three studies (6%) that described the perspectives or experiences of patients in acute care [13]. Evidence suggests a negative difference between patients' expectations of compassionate nursing care in hospital, and what they experience [21]. Patients' experiences in medical and surgical wards have highlighted that providing compassionate care takes time and commitment, but that even fleeting moments can establish a connection between a patient and nurse [22]. Compassion is recognised as a universal care concept which is uniquely perceived, enacted, and received through each patient's and HPC's individual lens which incorporates language, culture, and ethnic background [23]. Recommendations have been made regarding contemporary nursing practice that promotes compassionate values in recruitment and selection, education, organisational culture, mentorship, leadership, systems, and processes [24].

To date, no studies have compared and contrasted patients' perceptions of PCCC between professions in acute care wards, or examined factors such as the influence of patient disability, ward type, or length of stay (LOS).

## 2 Methods

### 2.1 Study Aim and Design

This study aimed to explore patients' perceptions of PCCC to inform co‐design of an intervention to enhance PCCC in general medical and surgical wards. Study objectives were:

i. To gather patient perceptions of PCCC including identifying areas of concern and strengths to build on.
ii. To compare compassion scores by ward type, patient demographic characteristics, LOS, and HCP category.
iii. To develop key messages for staff regarding patient and carer perceptions of PCCC.

The study used a concurrent mixed methods design, in which participants were provided the option to complete either a questionnaire regarding their experiences with medical, nursing, and allied health services, or participate in qualitative interviews. This design was pragmatic for the setting and facilitated investigation of the multifaceted and complex phenomenon of PCCC in healthcare [25]. Elevating the patient voice when describing PCCC is widely recognised as essential [3, 13, 16] and is in concert with mandated partnering with consumers accreditation standards, legislation, health policy, and public expectations [1, 26, 27].

This research was conducted with ethical approval [AM/2024/QMS/108721] and all participants provided written informed consent. Due to the use of patient interviews, the COREQ reporting checklist was used to guide reporting [28]. The GRIPP2‐SF [29] guided reporting for the co‐produced aspects of the study.

### 2.2 Setting

The study was conducted on a general medical and a general surgical ward of a medium sized (250 bed) metropolitan general teaching hospital in South‐East Queensland, Australia from September 2024 to March 2025. The hospital served a diverse community with more than 31% of patients being born overseas, more than 23% speaking a language other than English at home, 2.8% identifying as Aboriginal and/or Torres Strait Islander, and 20% with a disability [30]. The medical ward consisted of 28 beds with an average length of stay of 3.4 days. The surgical ward consisted of 28 beds, with an average length of stay of 2.8 days.

### 2.3 Participant Selection

All adult patients who had been on either the medical or surgical ward for three or more days were eligible to participate. Either the patients or their delegate provided informed consent. All participants had to be clinically stable, not have high levels of pain, and have no imminent diagnostic or interventional procedures scheduled. Where a patient had a cognitive impairment, they were excluded from completing the written questionnaire but were eligible to participate in an interview with carer consent and support. An interpreter or family member assisted where the patient did not speak fluent English.

Participants were initially recruited through convenience sampling. When nearly two‐thirds of participants had been recruited, purposive selection was used to achieve balanced representation across key participant characteristics ensuring diversity within the sample. As the study was exploratory, a sample of 30 patients from each ward (total of 60) was considered by a consultant biostatistician to be sufficient to estimate measures of central tendency and variability using the questionnaire, without the results being dominated by individual outliers. To capture data that would tell a rich and multifaceted story about patients' experiences of PCCC [31], 10 participants from each ward (total of 20) were recruited for the interviews.

### 2.4 Data Collection

The questionnaire consisted of the Sinclair Compassion Questionnaire (SCQ) [32] and additional questions regarding the patient experience. The SCQ is considered the ‘gold standard’ compassion measure for patients who have been in hospital for more than 7 days, but can also be used for shorter stays [32]. It consists of 15 items with each item rated using a 5 point Likert scale (strongly disagree, disagree, neutral, agree, strongly agree) across the following compassion domains: Attending to needs (2 items); Seeking to understand (2 items); Relational communication (7 items); Relational space (2 items); and Virtuous response (2 items) [12]. Individual responses to each item were coded numerically 1–5, tallied, and then averaged to create the overall score for each questionnaire. Higher scores are considered indicative of a greater experience of compassion [32]. After completing the SCQs for each professional group, participants were invited to respond to five open‐ended questions exploring the person's perception or experience of PCCC during the admission (See Box 1). The researcher was available to write down participants' scoring and open‐ended question responses if requested. Participants who completed the questionnaire did this three times—considering their interactions with doctors, nurses, and allied health separately.

Box 1: **Qualitative questions**

**Table:** 

1. Can you tell me what person‐centred compassionate care means to you?
2. Could you describe an instance where you experienced person‐centred compassionate care?
3. What things affect the ability of healthcare providers to offer person‐centred compassionate care?
4. How can we best support person‐centred compassionate care and communication with people from diverse backgrounds or cultures?
5. Are there any other comments you'd like to offer about the care you received in hospital?

Participants who took part in only the qualitative interviews were asked semi‐structured questions exploring the same five topic areas included at the end of the SCQ (Box 1). All interviews were undertaken by experienced qualitative researchers (J.H. and R.C.). An inquiring, conversational approach was adopted during the interviews to explore participants' perspectives and experiences of PCCC during their hospital admission. Interviews ranged from 13 to 29 min with an average of 15 min. Three interviews were undertaken with carers/family members with or on behalf of patients with cognitive impairment.

### 2.5 Data Analysis

All demographic and SCQ data were entered into Excel, with inferential statistics run in SPSS version 29. Independent data quality checks were completed. There were only three (1.8% of values) missing responses and there was no pattern, hence this can be considered to be random, and the score was calculated using the available data. The total cohort data set (combined SCQ scores for 168 questionnaires) was found to be negatively skewed (SCQ combined skewness −1.357, SE = 0.187) and analysis of each subgroup group confirmed moderate to high levels of negative skewness in all groups (SCQ Medical skewness = −1.279, SE = 0.309; SCQ Nursing skewness = −1.692, SE = 0.309; SCQ Allied Health skewness = −0.834, SE = 0.343). Therefore, measures of central tendency were reported as median and standard error of the mean (SEM), and non‐parametric statistics were run for repeated measures (Kruskal–Wallis) and independent group (Mann–Whitney *U*) comparisons. Acknowledging that multiple comparisons were planned within each professional group (exploring differences by ward, gender, cultural background, disability, and LOS), a more stringent adjusted alpha of 0.01 was adopted for significance to minimise risk of Type 1 error.

Data from the open‐ended questions completed after the SCQ, were transcribed from paper forms by J.H. and R.C. in preparation for analysis. Interviews were digitally recorded and transcribed and checked for accuracy by R.C. or J.H. Transcribed qualitative data from the interviews, and open responses to questions at the end of SCQ were uploaded to NVivo Pro v12 [33], and analysed together using the six‐stage reflexive thematic analysis process [31]. This allowed for the examination of different perspectives and provided flexibility to highlight unanticipated insights. To maximise trustworthiness [34] two HCP authors (R.C. and J.H.) independently coded four interviews in NVivo Pro v12 [33], compared codes and revised where needed. J.H. coded the remaining data. R.C. and J.H. then reviewed codes and developed preliminary themes. R.C. mapped codes to preliminary themes in NVivo and downloaded to an Excel spreadsheet. Preliminary themes with all data were sent to a consumer co‐investigator (A.M.) and two more HCP investigators (E.W. and J.N.). Themes were refined over a 1.5‐hour meeting with this group and finalised in a subsequent meeting that included consumer F.E‐H. Due to scheduling issues, RC met with the surgeons (S.N., E.D.) separately to confirm themes. Other trustworthiness approaches included maintaining data familiarisation notes, review of research meeting summaries, and ongoing team reflection [31]. A contiguous approach to presenting data was planned [25].

In accordance with a concurrent mixed methods design, the intent of integration was to compare or match the results, by merging them in a joint display [35]. Analysis of the quantitative and qualitative data was performed separately. As the quantitative analysis resulted in a series of SCQ scores, integration involved comparing the SCQ domains underpinning these scores with the qualitative findings. The results are presented in a joint display, along with a narrative summary.

Following data analysis, the research team met twice to review the data to co‐create key messages to staff using the patient voice under each theme.

### 2.6 Patient and Public Involvement, Positionality, and Reflexivity

This co‐produced study included two consumer co‐investigators (A.M., F.E‐H.) from inception, throughout the research cycle. Study planning, progress, and data interpretation presentations with two‐way dialogue occurred five times with the hospital Consumer Advisory Council. Additionally, approximately 70 individual staff and consumers either at individual meetings or though consultation with four other hospital level committees were consulted regarding study findings. Box 2 includes the benefits, challenges, and influence of the consumer partnerships on the current study. A.M. was a consumer co‐investigator who bought disability lived experience due to his long‐term health conditions and numerous hospitalisations. He had been a co‐investigator in a study regarding a hospital in the home service and had been a consumer partner at the hospital for 5 years. He received remuneration for his involvement. F.E‐H. had been a consumer partner with the hospital and health service for over 10 years. She brought culturally and linguistically diverse expertise and was an academic with research interests in social inequality across gender, faith, and culture. She declined remuneration.

Box 2: **Benefits, challenges, and influences of the consumer partnerships on the study**

**Table:** 

**Benefits:**
Privileging the patient voice was emphasised throughout the study.
Participant information sheets and consent forms were substantially enhanced and written in plainer language.
Consumer positionality was included in data analysis with a focus on disability and cultural diversity.
Rich actionable insights were developed from the qualitative data.
The consumer co‐investigators emphasised several implications for the discussion which were not identified by the HCP.
Several HCP research team members acknowledged substantial new learning because of consumer partnerships which strengthened this study and built capacity for the future.
**Challenges:**
Limited availability of one consumer required additional time to ensure their perspectives were included.
Additional requirements from the ethics committee including consumer agreements required extra time.
The administrative burden of consumer remuneration for the PI, hospital finance, and the consumer.
Partnering with consumer co‐investigators with different routines to HCPs added to the complexity of arranging meetings.
**Influences:**
Including the rich actionable insights for HCP from the thematic analysis was novel and strengthened study translation.
Participant information sheets and consent forms were more health literate and patient‐friendly.
Consumer partnering capabilities were strengthened across the research team.
Consumer co‐investigators added credibility to whole research cycle, especially the findings and recommendations.
The Consumer Advisory Council provided the organisational governance and support to the study. This was crucial in obtaining ethics and research governance clearance, mobilizing leadership support, translating the findings into action, and developing future directions for the next phases of the study.

All team members were committed to improving PCCC within the study setting and bought this lens to all study activities. The PIs were a Director of Occupational Therapy (R.C.) with research interests including partnering with consumers in quality improvement and research; and a Senior Nursing Research Fellow (J.H.) with a conjoint appointment between the hospital and a university, and research interests including decision‐making at the end of life. The study mentor (E.W.) was an experienced health services researcher with no clinical involvement on the wards involved. J.N. was an occupational therapist and manager of the health service consumer partnering unit with research interests in partnerships for healthcare governance and communication in cancer care. E.D. was the Director of General Surgery at the hospital and had research interests in breast cancer and gastrointestinal surgery. S.N. was the health service surgical stream lead and a general surgeon. He provided the impetus for the study by advocating for change at the hospital Consumer Advisory Council following his personal healthcare experience.

Reflexivity was considered an ongoing and collective process throughout the study. Team members brought diverse professional, personal, and experiential perspectives that shaped how PCCC was understood and interpreted. The team's commitment to improving PCCC within the study setting, sensitised them to examples of compassionate care but also required conscious reflection on potential assumptions regarding what compassionate care should look like in practice. Regular structured meetings provided opportunities to critically examine interpretations, challenge assumptions, and explore alternative explanations for findings. These processes were guided by co‐production principles emphasising shared decision‐making, reciprocal relationships, reflexive dialogue, and recognition of the different forms of expertise represented within the team. [36, 37].

## 3 Results

### 3.1 Participant Characteristics

Sixty (60) participants completed the SCQ and provided written responses to the qualitative questions. An additional 20 participants took part in an interview. The characteristics of the 80 participants were diverse as shown in Table 1. Approximately half the participants were on the surgical ward (*n* = 39;51%) and half on the medical ward (*n* = 41,49%). Median length of stay was 6 days (range=3‐ > 50 days).

**Table 1:** Participant characteristics.

Participant Characteristic		Sinclair Compassion Questionnaire		Interviews
		Doctors & Nurses *N *= 60 for both (*n*, %)	Allied Health *N *= 48 (*n*, %)	All disciplines *N *= 20 (*n*, %)
Ward	*Surgical*	32 (53.3)	29 (60.4)	10 (50.0)
	*Medical*	28 (46.6)	19 (39.6)	10 (50.0)
Gender	*Woman*	31 (51.2)	25 (52.1)	9 (45.0)
	*Man*	29 (48.2)	23 (47.9)	11 (55.0)
Language other than English at home	*Yes*	8 (13.1)	4 (8.3)	7 (35.0)
Aboriginal or Torres Strait Islander	*Yes*	8 (13.1)	4 (8.3)	2 (10.0)
Diverse cultural or ethnic background	*Yes*	18 (30.0)	12 (25.0)	9 (18.0)
Disability	*Yes*	19 (31.2)	16 (33.3)	7 (14.0)
Lived experience of mental ill‐health	*Yes*	18 (30.0)	14 (29.2)	6 (12.0)
Length of stay in days		M = 5.5 (IQR, 4)	M = 9.0 (IQR, 5.5)	M = 6.0 (IQR, 2)
Age in years		M = 63.5 (IQR, 33.5)	M = 69.0 (IQR,37.5)	M = 74.0 (IQR,28.75)

### 3.2 Sinclair Compassion Questionnaire

Sixty (60) SCQ questionnaires were completed regarding both doctors and nurses. Only 48 sets of SCQ data were collected for allied health as 12 participants could not recall having interactions with this professional group. Collectively, these 168 SCQ responses yielded a median score of 4.5 (SEM = 0.05). Fewer than 20 per cent (17.9%) of the SCQ scores were less than 4, with more than half (51.2%) scoring 4.5 or higher. Results indicated that overall, study participants perceived that they experienced high levels of compassion from HCPs during their hospital admission.

**Figure 1:**
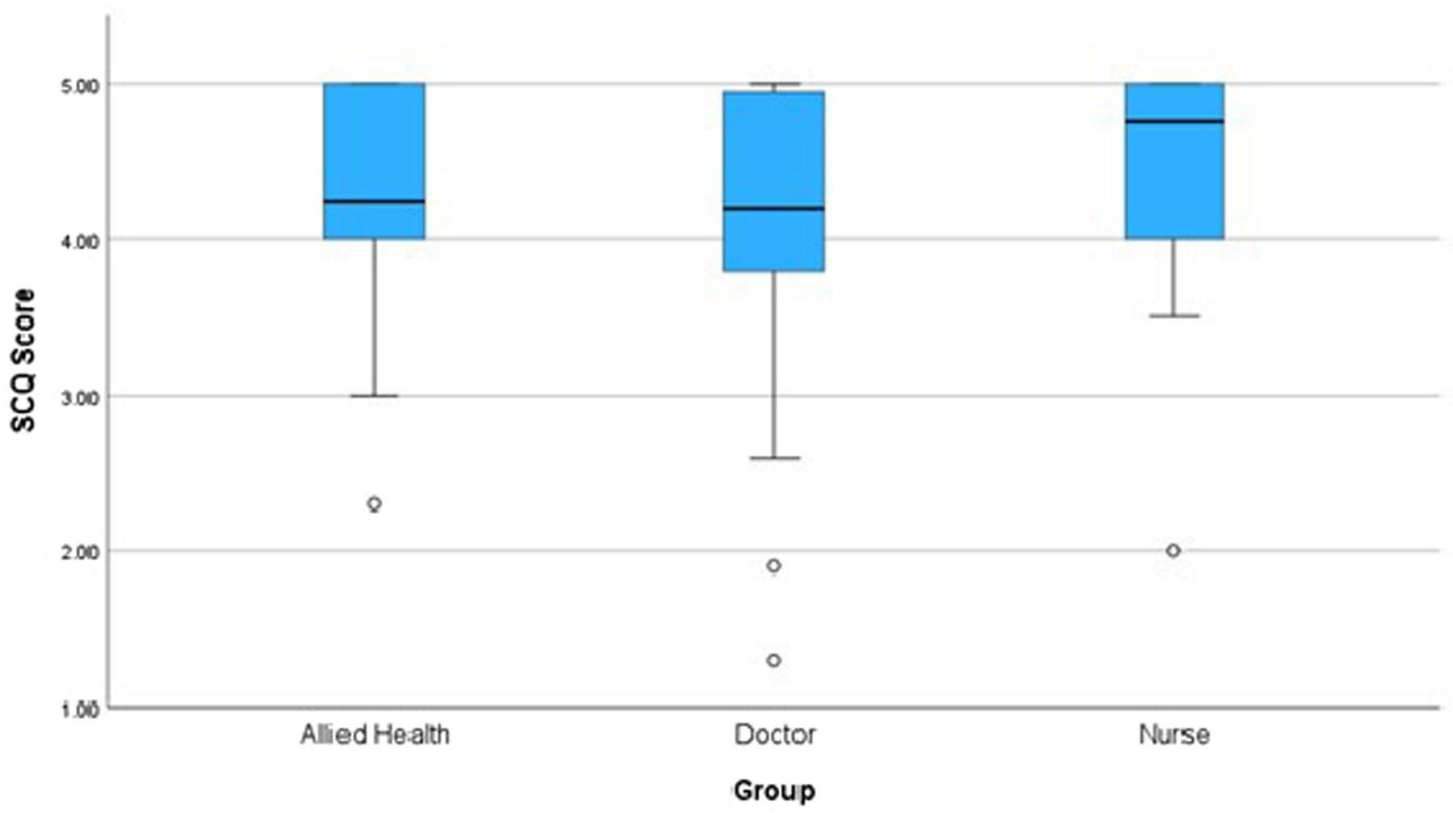
Distribution of Sinclair Compassion Questionnaire (SCQ) scores for the three professional groups.

Data were examined to see if there was any difference in SCQ score depending on professional group. Analysis revealed no statistically significant differences (*p* = 0.066) with overall high scores observed for all groups (Doctors: median = 4.2, SEM = 0.10; Nursing: median = 4.75, SEM = 0.07; Allied Health: median = 4.25, SEM = 0.09) (Figure 1). Data for each professional group were also explored to see if there was any difference in SCQ scores depending on ward (medical or surgical), gender, cultural background, disability, and LOS. Results revealed no significant difference (*p* > 0.01) for any variable except gender (*p* = 0.003) where females rated allied health staff as significantly higher on SCQ (median = 4.9, SME = 0.1) than males (median = 4, SEM = 0.15). No gender differences were observed for doctors or nurses. No analysis could be conducted for language other than English and Aboriginal and/or Torres Strait Islander background due to insufficient numbers.

### 3.3 Qualitative Findings—from ‘Little Things’ to ‘Seagulls’

From ‘little things’ to ‘seagulls’ was a phrase co‐created by the research team to encapsulate the perspective of many participants as per these indicative quotes: ‘… the kindness would be like, you know, like just doing those little things… helping me do something.’ (P73‐IV) And, ‘Doctors were like a flock of seagulls. They fly in, squark at you (as a group) and fly off.’ (P34‐Q)

**Figure 2:**
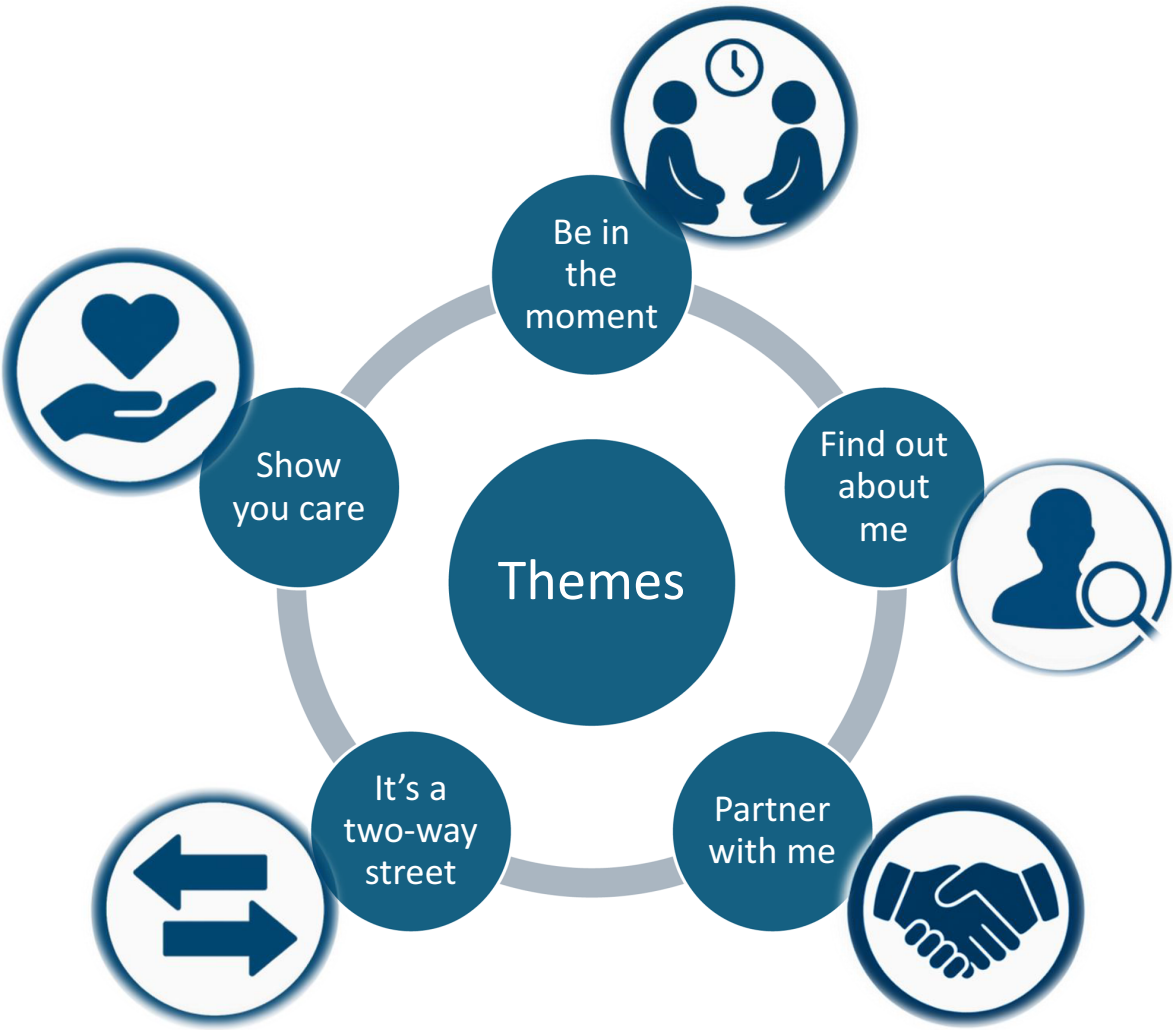
Summary of themes.

There were five qualitative themes (Figure 2) which are detailed below. The participant number is provided beside quotes and indicates if the data were from an interview (IV) or from the open‐ended questions after the SCQ (Q).

Theme 1: Be in the moment

In this theme, the importance of HCPs taking the time to be attentive, focussed, and actively listen to create a safe space was highlighted. Many patients and carers talked about knowing that staff had limited time but wanting them to slow down, and to be focussed, rather than distracted. In contrast, feeling that the HCP is present, actively listening, and in the moment was reported to make patients feel safe. A patient provided this advice for staff, ‘Time is important and if you have limited time, presenting yourself in a way where you're approachable at this moment….’ (P74‐IV) The importance of full attention was discussed many times as essential to PCCC, as was active listening by HCPs. For example, a patient said ‘… they would ask a question, I would ask a question back and you could see that they were actively listening to what I was asking rather than just hearing some keywords and then responding back to me.’ (P75‐IV)

The issue of computers distracting nurses from focussing on the patient was raised. One participant observed ‘the computer [is] getting in the way of doing the nurses' work’ (P27‐Q), whereas another found nurses referring to the computer quite distressing:

In my circumstance, the initial response [from the nurse] is ‘I'm looking at the computer, everything's under control’. Yeah, to somebody that's going through a panic attack, that's not what I need to hear. (P67‐IV)

Theme 2: Find out about me

In Theme 2, the importance of HCPs getting to know the whole person in a non‐judgemental way as an individual, inclusive of family, cultural background, age, disability, and living situation was emphasised. A patient noted, ‘Person‐centred to me means the individual. So, to me this means that compassionate care to that individual as they present, not the problem they present with. Everyone is different. Everyone needs to be heard.’ (P12‐Q) Many participants provided examples of culturally and linguistically diverse staff and patients contributing to positive experiences. The notion of understanding the journey that led to the patient's hospitalisation was reported many times, ‘… they need to get a better understanding of the circumstances of how patients entered the premises…. they make the assumption that, oh, well, he's only just reported here today.’ (P67‐IV) Additionally, participants talked about wanting to be seen as a human being, not just a number, or a ‘… another piece of meat.’ (P12‐Q) It was suggested that HCPs treat patients like they would their own family. The need to meet emotional and mental health needs including past trauma such as loss of a child or depression and anxiety, was emphasised: ‘If your brain is not right, everything goes out the window. Support for mental health is just as important as physical health.’ (P58‐Q) The feeling of vulnerability and ‘… not [being] at the top of their game’ (P67‐ IV) was an additional message that participants wanted HCPs to hear.

Theme 3: Partner with me

Theme 3 highlighted that patients wanted to feel connected with HCPs involved in their care but also to choose the level of partnership. To do this, they needed to understand the information provided. Clear, appropriate communication was important. Explicitly acknowledging the partnership was recommended:

But she [the doctor] made it quite obvious that the exercise that she and I'd been through was a partnership.… She said, ‘I've got as much going on this job as you have.’ And that was a very good way of her putting it…. She was not just an interested bystander, you know. She was an active participant. (P71‐IV)

Core to this active partnership was the imperative of explaining things in a way that the patient and family could understand to support decision‐making. Participants gave examples of groups of doctors coming and talking amongst themselves in technical language and not involving them. For example, ‘Some staff don't break down the medical terminology in plain English, so I feel left out of the conversation.’ (P50‐Q) In some instances, patients were excluded from the partnership because HCP did not take the time to listen, making it difficult to raise concerns. One patient reported, ‘[my] first two admissions, doctors, like seagulls, fly in as a group, squark and don't listen. Admission two, they couldn't get me out fast enough, even though I had concerns.’ (P34‐Q)

Poor or fractured communication due to staff changes impacted patients and their families in a variety of ways, including their discharge planning, medication changes, cancelling of surgery while the patient continued to fast, and lack of information about modified diets. These issues were exacerbated for patients with cognitive impairment and/or who did not speak English. A patient talked about, ‘… information asymmetries, meaning constant cycling of nurses and doctors throughout my 5 days which undermine my trust that each person had the context about my situation.’ (P39‐Q) Another patient talked about feeling like they had a relationship with staff when they remembered them on subsequent days, and others appreciated being ‘kept in the loop’ and having their questions answered in a way they could understand.

Theme 4: It's a two‐way street

In Theme 4, participants recognised the challenges faced by HCPs and the limitations of available resources. They acknowledged that most HCPs strive to provide PCCC, but there are instances where this standard is not met. Caring for patients with more complex or demanding needs was seen as challenging. It was acknowledged that staff could not control all aspects of the healthcare experience due to factors such as budget constraints, ‘[I] Waited for 6 h on a trolley but that feels like a governmental thing not a staff thing.’ (P22‐Q) Though conversely, another patient also talked about the importance of ‘Having empathy and thinking about what the person needs and wants, as opposed to what the system needs and wants.’ (P47‐Q) There was also acknowledgement that staff were often impacted by other patients, and that managing patients with challenging needs impacted both staff and other patients, ‘My first night, it was clear that my nurse had a lot on her plate, and took a while to help me, but I completely understood ‘cause she was next door getting yelled at by a patient.’ (P71‐IV)

Recognising the pressures and competing demands on HCPs including, ‘…buzzers going off left, right and centre’ (P71‐IV), was a common topic:

The pressures of their jobs. Plain and simple. They've got requirements that they've got to meet… the amount of patients they have every day…. that makes it difficult for them to be able to sometimes be person centric…. (P16‐Q)

Several participants noted that most staff were compassionate and caring but there was always, ‘… a bad egg in a dozen’ (P28‐Q), and ‘there was one person who had it in for me. I couldn't communicate because they weren't nice. Maybe I rubbed them up the wrong way.’ (P29‐Q)

Theme 5—Show you care

The last theme illustrated that small acts of kindness, including gentle touch, appropriate humour, and compassion, demonstrated respect and helped patients feel their needs were a priority. In one instance, a patient was provided a breakfast they did not want. They reported, ‘… and I said, “could I have porridge, please?” I had it in 5 min. Hot.’ (72‐IV) Another example was staff attaching a patient arm band to a cuddly toy that belonged to a cognitively impaired patient. Being made to feel like a true priority contributed to participants' positive experiences:

Made to feel not so much special, but you're important and the connection between you and the staff is authentic… even though it's been painful, it's been a very… not delightful, but a very caring feeling every day. (65‐ IV)

The son of a cognitively impaired patient reported, ‘… the tone that they speak to her is very good. That's why she feels it, even though she can't speak English.’ (P64‐IV) Humour was reported to be critical to PCCC, ‘I've got a good sense of humour… laughter is the best medicine. Yeah. If you can make somebody laugh, it makes them feel better.’ (71‐ IV) Conversely, it was viewed negatively when nurses had ‘… personal conversations in front of patients. Lots of laughter while ignoring patients. [I do not] appreciate nursing staff entering [my] space without permission and sitting on [my] bed.’ (P36‐Q) Another negative experience for patients was when doctors were, ‘… very practical, and solution focussed…. lack of me being at the centre of the team.’ (P51‐IV) Feeling respected was core to a positive experience.

### 3.4 Integration of Findings

Quantitative results indicated that overall, participants experienced high levels of compassion from HCPs during their hospital admission, with no statistical difference between professional groups or wards. Qualitative findings revealed that despite patients' overall satisfaction with PCCC, how it is experienced can be variable. These findings were compared in the joint display (Supporting Information S1: File 1).

Participants described compassion as fundamentally relational, experienced through HCPs being present, attentive, and authentically engaged. The themes *Be in the moment* and *Find out about me* highlighted that patients felt cared for when clinicians listened, sought to understand them as individuals, and acknowledged their unique circumstances, values, and needs. Compassion was diminished when interactions felt rushed, impersonal, judgemental, or dismissive. These findings align with the SCQ domains of *Relational space, Relational communicating, Seeking to understand*, and *Virtuous response*, confirming that compassion requires recognising and responding to an individual's unique needs and circumstances.

Participants also understood compassion within the realities of busy healthcare environments. Through the *Two‐way street* theme, they acknowledged workforce pressures and competing demands, yet still expected respectful, person‐centred interactions. Compassion was further experienced when patients were treated as partners in care (*Partner with me*) and when caring intentions were translated into visible actions (*Show you care*), such as providing comfort, maintaining dignity, and responding to individual needs. These themes reflect the SCQ domains of *Relational communicating, Attending to needs*, and *Virtuous response*, confirming that compassion is enacted through partnership and responsive care.

Collectively, these findings indicate that compassion is both a way of relating and a way of acting: it is co‐created through person‐centred relationships and realised through responsive behaviours that demonstrate understanding, respect, and genuine concern for what matters most to patients.

### 3.5 Co‐Developed Patient Advice to Staff

Incorporating the study findings, the co‐developed patient advice to staff relating to each theme is displayed in Table 2. These provide staff with clear messages derived from the patients' experiences to help enhance PCCC.

**Table 2:** Co‐developed patient messages to staff.

Theme	Key messages to staff
Be in the moment	Take the time Be attentive Be focussed, not distracted Really listen to me Your attention makes me feel safe
Find out about me	I am a whole person Understand my physical and mental health My journey and story are important Don't judge me Understand my culture, age, disability, where and how I live I feel vulnerable
Partner with me	I want a partnership to the level I choose Connect with me, form a relationship Communicate so I understand and can decide Include my family Inadequate team and other communication greatly affect me
It's a two‐way street	I am understanding and make allowances for staff challenges Staff do their best within resources Some staff don't provide person‐centred compassionate care, but most do I understand that some patients are more challenging for staff
Show you care	Acts of kindness mean a lot—touching, humour, gentleness, hugs, food Make me feel like a priority Help me with daily needs Reassure me and show respect

## 4 Discussion

This co‐produced study adds essential new information regarding the perceptions of patients from diverse backgrounds in acute hospital general medical and general surgical wards regarding PCCC. Prior to this study, privileging the patient voice regarding this topic in acute hospitals has been limited [16, 38]. To the authors' knowledge, this is the first research to use the Sinclair Compassion Questionnaire (SCQ) in the acute general medical and general surgical inpatient context which is valuable as scores can vary across care type [12]. Overall, patients indicated that they experienced high levels of compassion on the SCQ with 51.2% of respondents scoring 4.5 (out of 5) or higher. It is essential to share positive feedback such as this with staff and the community to recognise and celebrate the positive aspects of care [39]. However, when asked, patients offered many recommendations to enhance care. This apparent inconsistency highlights the value of mixed methods research and may indicate a degree of ceiling effect for the SCQ in acute general and medical wards. This finding is novel and indicates that further research using the SCQ in acute care is required.

Literature which supports the use of quantitative Patient Reported Experience Measures (PREMS) for service accountability, warns that these measures are a means to an end and not an end in itself [3]. Organisations need to analyse and use patient experience data to identify gaps and to develop quality improvement directions [27]. Thus, including a qualitative aspect in measuring patient experience is a strong recommendation from this study. Qualitative data ensures that organisations know what matters to patients [2, 3]. Other research has also found that patients often reported specific stories, such as the seagull instance, that can facilitate individual and organisational learning to drive positive change [39]. Improving PCCC enhances the patient experience and outcomes [40], and importantly may help mitigate HCP's stress of conscience in hospital settings and influence staff wellbeing and retention [7, 11].

Similar to previous research in other care settings, there were no statistically significant differences in the SCQ scores for patients with culturally diverse backgrounds [12]. Several participants referred to the cultural diversity of patients and staff. This reflected the demographics of the local area and may have meant that the hospital was welcoming of cultural diversity, provided access to interpreters, and prioritised appropriate learning and development as recommended by others [13, 23]. This may not be the case for other hospitals. For gender, the only statistically significant difference was for females who rated allied health staff as more compassionate. Interestingly, there were no statistically significant differences in SCQ scores between ward type, LOS, between medical, nursing, and allied health professions, or for patients who identified as having a disability.

The authors are not aware of any other research making these direct comparisons using the SCQ. The ability of the SCQ instrument to detect subtle differences between groups of HCPs, patient demographics, LOS, or ward type may have resulted in a ceiling effect in acute medical and surgical wards. Accordingly, non‐significant group comparisons should be interpreted with caution and viewed as more of a ‘temperature check’, than a definitive finding. A previous study exploring patient encounters with nurses, doctors, allied health professionals, and multidisciplinary teams reported that empathetic and compassionate care from a variety of healthcare providers was important [19]. It may be possible to distribute compassion among members of the multidisciplinary team, making PCCC both an individual and team endeavour [41]. The high SCQ scores across all professions in this study, supports the notion that PCCC can be shared between HCPs to modulate instances when compassion does not meet patient expectations. This is an important finding.

A strength of the study was that the research team, inclusive of consumers, co‐produced five themes with key messages to staff to drive positive change. The themes were action oriented, and encompassed the dynamic nature of PCCC as responsive and proactive [13]. Firstly, *Be in the moment* highlighted the need for staff to actively listen, and to be attentive and focussed, as this helped patients feel safe. The value patients place on staff taking the time to listen [13, 20], and on calm and unhurried compassionate dialogue with HCPs has been reported elsewhere [5]. Even the briefest of encounters can positively impact patient outcomes which is encouraging in increasingly time‐pressured healthcare environments [16]. The importance of HCPs recognising this need to bring their full presence as a conscious choice is essential when providing comfort and care [42]. The research team discussed that the nature of a teaching hospital may have impacted on patients' perception of the focus of medical staff as they juggle patient care and education roles. This requires further investigation.

The *Find out about me* key message encapsulated the essence of person‐centred care which recognises that people are unique and their health goals result from their context, what matters to them [43], and their life story [44]. This current study identified that patients felt vulnerable and that staff could be judgemental, overlooking their journey, especially their mental health. Understanding the individual patient and their whole situation before moving to action is critical when responding to suffering [38, 45] as is the importance patients place on HCP being respectful [20] and nonjudgemental [19].

The third theme, *Partner with me*, highlighted patients' need for a partnership to the level they choose, authentic connections with HCPs, clear communication in a manner they understand, inclusion of family, and the impact of inadequate team communication. Communication skills are inclusive of listening, touch, body language, eye contact, and positive demeanour [16, 19]. Patients' engagement preferences are not universal and should be assessed at hospital admission [46] and ongoing [1]. It is critical that HCPs understand the dynamic nature of health literacy which may fluctuate due to ill‐health and the unfamiliar hospital environment [45]. The central importance of relationship development and the critical role of individual and team communication in PCCC are not new concepts [38, 44]. This study highlights the patient perspective.

*It's a two‐way street* emphasised that patients make many allowances for the pressures and challenges experienced by HCPs, including other patients with higher needs. This theme, along with *Partner with me* suggests that patients do not necessarily see themselves as passive recipients of compassionate care—a novel finding that has not, to the authors' knowledge, been well recognised in the literature. This contrasts with an earlier study which highlighted that even within significant time and resource constraints, patients still expected nurses to have time for and listen to patients [22]. The finding here concurs with the premise that seeking to mitigate patient suffering, and respond with compassion may be enhanced by structural, organisational, and HCP practices [14, 40].

The final theme, *Show you care*, demonstrated, in concert with the literature, that small acts of kindness are vital to the patient experience of healthcare [13, 19, 23, 47]. Patients wanted proactive help with their small daily needs in addition to routine healthcare [16]. Touch, including a hug and gentleness, were valued as reassuring and conveying a feeling of safety as noted by others [23, 42]. This study and others [19, 38] have highlighted the need for greater HCP and organisational value and recognition of the big impacts of seemingly small acts of kindness. The emphasis patients made on humour and providing food as comfort were findings not discussed in detail in the literature reviewed.

### 4.1 Limitations

This study has several limitations. Data were collected within a single hospital, which may limit the transferability of findings to other healthcare settings. Patients who were currently experiencing high levels of pain, or likely to attend a procedure were excluded, and this could influence perceptions of PCCC. Most patients requested assistance to complete the SCQ which suggested that they truly valued conversations about their experiences but were not confident to complete paper‐based forms. Whilst the research assistants who supported collection of the data were not involved in patient care on the study wards, there is still potential that this may have influenced patients to provide higher scores. The high scores on the SCQ may reflect a ceiling effect of the measure [48]. This requires further investigation in the acute hospital setting to ensure the tool is fit for purpose. Additionally, details of patients who declined to participate were not included. Participants scored their overall experiences against broad HCP categories which meant that factors such as years post qualification, type of allied health professional, and HCP demographics were not examined. Type of disability was not collected, and future research may be required to ascertain if this is an important factor.

In addition, most qualitative data were coded by a single researcher, potentially increasing the influence of individual interpretive perspectives during analysis. However, several researchers, including a consumer co‐investigator, reviewed preliminary themes against indicative data, and the full team finalised themes which supported trustworthiness of qualitative analysis. Additionally, the integration of quantitative and qualitative findings, together with regular team discussions regarding coding and interpretation, strengthened the overall rigour and credibility of the study.

Whilst this mixed methods study included qualitative written questions and interviews, participant observation or an ethnographic study with multiple data sources may have revealed more in‐depth insights into the patient experience [10].

### 4.2 Implications for Practice

Five actionable messages in the patient voice have been co‐developed and have direct implications for HCP practice regarding PCCC. In terms of research practice, this study has highlighted the benefits of co‐production with consumers to elevate findings to actionable insights which privilege diverse patient perspectives in improving healthcare [49]. The need to invest in qualitative analysis of PREMS data including links with researchers to ensure qualitative rigour [2] has been highlighted again by this study. Several papers have discussed the need to invest in evidence‐based staff learning to enhance PCCC [13, 44, 50, 51]. However, it is unclear which approach is more efficacious but all should be experiential with critical self‐reflection [43, 44, 51, 52] and be encouraged by organisational leadership and a supportive organisational culture [3, 44, 50]. The next step for this current research team is to partner with patients, consumer partners, and HCPs to co‐design an intervention to implement the co‐developed recommendations discussed here.

## 5 Conclusion

This co‐produced study amplified the voices of a diverse patient group and generated actionable insights into PCCC across acute medical and surgical wards. While SCQ scores were consistently high across disciplines, ward types, length of stay, and patient characteristics, qualitative feedback highlighted opportunities for improvement, underscoring the value of including qualitative components in PREMs. Patients emphasised the importance of being present, understanding the whole person and their journey, partnering at the patient's preferred level, recognising that patients appreciate service pressures, and acknowledging that small acts of kindness have a profound impact on care experiences.

## Author Contributions

**Ruth Cox:** conceptualization, funding acquisition, data curation, formal analysis, investigation, methodology, project administration, resources, validation, writing – original draft, writing – review and editing. **Jayne Hewitt:** conceptualization, investigation, funding acquisition, writing – original draft, methodology, validation, writing – review and editing, formal analysis, data curation, resources. **Emilia Dauway:** conceptualization, investigation, methodology, project administration, validation, writing – review and editing. **Alex McConnell:** conceptualization, methodology, validation, writing – review and editing, and formal analysis. **Jodie Nixon:** conceptualization, formal analysis, investigation, methodology, validation, writing – review and editing. **Faiza El‐Higzi:** conceptualization, investigation, methodology, validation, writing – review and editing. **Sanjeev Naidu:** conceptualization, investigation, methodology, validation, writing – review and editing. **Elizabeth Ward:** funding acquisition, writing – review and editing, methodology, validation, formal analysis, conceptualization, and investigation.

## Patient or Public Contribution

This co‐produced study included two consumer co‐investigators (A.M., F.E‐H.) from inception, throughout the research cycle. Additionally, study planning, progress, and data interpretation presentations with two‐way dialogue occurred five times with the hospital Consumer Advisory Council.

## Conflicts of Interest

The authors declare no conflicts of interest.

## Supporting information


Supporting File

## Data Availability

Summary data that support the findings of this study are available upon reasonable request from the corresponding author.
