# Supplementary material for: From ‘Little Things’ to ‘Seagulls’: A Mixed Methods Exploration of Patients' Experiences of Person‐Centred Compassionate Care in General Medical and Surgical Wards
Source: Health Expect. 2026 Aug 30;29(5):e70844. doi: 10.1111/hex.70844 (PMC13527410; doi:10.1111/hex.70844)
Supplement: Supplementary file 1 [file HEX-29-e70844-s001.docx]

| **Compassion Domains** | **Themes** | **Exemplar Quotes (demonstrating compassion)** | **Exemplar Quotes (demonstrating the need for improvement)** |
| --- | --- | --- | --- |
| **Relational space Relational communicating** **Attending to needs** | **Be in the moment** Highlights the importance of HCP taking the time to be attentive, focussed, and actively listen to create a safe space. | Just how you're carrying yourself in that moment, how you're presenting yourself to the patient. The nature of your responses- are you willing to answer a question that's…not directly related to the signs and symptoms. [P74 Interview] | Could feel a bit more listened to in terms of medication updates. My file was old, and I was telling them my updated meds but they didn't listen. [P22 Survey Qual Response] |
|  |  | When the nurses had to stay with me while I had an IV, but they took the time to talk to me. Not just stare at the wall. [P19 Survey Qual Response] | Gotta be in meetings. You gotta be doing your, you know. Paperwork. So, you know, if everything's compounding on. You don't have enough time to to sit back and actually dedicated to going well. What you know, what is this patient actually needing? [P78 Interview] |
| **Seeking to understand Virtuous** **response** | **Find out about me** The importance of getting to know the whole person in a non-judgemental way as an individual, inclusive of family, cultural background, age, disability and living situation. | They couldn’t really speak Mandarin, but you know, they hand gesture trying to make all the people understand what they're really trying to tell, though, you know, the language barrier there, you can feel that’s been caring. [P79 Interview] | No, because I thought, like, you know I was just like she probably had a bad day. But when it was constantly happening, that's when I was like, OK, now she's... I think she just thinks I'm too young to be in this ward and that I shouldn't be here because I'm on my phone. I was like, I'm on my phone. But I’m on my phone because this is the way I contact my family. I'm on my phone because this is the only thing I could do because I can't move my body. Like, do I need to explain more? But like, yeah, she... I think she just didn't like me because I am young. [P73 Interview] |
|  |  | I think just making sure that the patient is comfortable mentally and physically. You know, basically feeling comfortable under the circumstances. And I guess obviously compassion is the important word is being able to understand that the patient is undergoing a pretty stressful situation. [P71 Interview] | They're making decisions on people without knowing who they are. They've got the details and the bloods and all the rest of it. So, they can fix the physical symptoms. But yeah...I think they've lost contact with, with who they're working with. [P67 Interview] |
| **Not represented** | **Two-way street** There are challenges faced by HCP, including resource constraints, and caring for patients with complex or demanding needs. | *The pressures of their jobs... they've got Between 3 and 7, people that are pressing their dinger and saying “Come and help me. Come and help me. Come and help me.” And they've got to juggle all of that. And that makes it difficult for them to be able to sometimes being person centric and because they under so much pressure and that can be, that can be a trigger for some people. [P75 Interview]* | *There was one person who had it in for me. I couldn't communicate because they weren't nice. Maybe I rubbed them up the wrong way [P14 Survey Qual Response]* |
|  |  | *I would understand their workload may be highly impactful. My first night, it was clear that my nurse had a lot on her plate, and took a while to help me, but I completely understood ‘cause she was next door getting yelled at by a patient.  [P29 Survey Qual Response]* | *Their personality. I can tell when someone does not want to be working in a clinical environment [P6 Survey Qual Response]* |
| **Relational space Virtuous response Seeking to understand** | **Partner with me** Patients want to feel connected with the HCP involved in their care, but also to choose the level of partnership. Clear appropriate information is imperative to achieve this. | If you need to talk, they'll sit and talk to you. You know, they've got work to do. But you come first. That's it to me, that you're worth it to them. That's what you want, people that you want, you want to feel like you're worth it to, to staff. [P63 Interview] | I found that they were lovely but still don’t listen to patient's expertise of own health. It feels like nurses’ underestimate patient's experiences of pain. [P36 Survey Qual Response] |
|  |  | She says, “Are you OK?” Yeah. Yeah, that's her approach. How are you, [patient first name]? Are you OK? The first thing she asks . OK. She, she shows very obvious concerns for my well-being. Which is good. [P71 Interview] | Sometimes the Drs take too long to tell me what is going on and give me conflicting information about my diagnosis. [P42 Survey Qual Response] |
| **Attending to needs Virtuous response Relational communicating** | **Show you care** Small acts of kindness, including gentle touch, appropriate humour and compassion, demonstrated respect and helped patients feel their needs were a priority | She actually had given him a haircut and shaved him. So, they had actually developed a closeness enough that he would sit and allow her to actually cut his hair. And shave his beard. [P78 Interview] | When I'm trying to get into bed, not showing you help, or not even covering you up properly, or not covering you up at all, actually. [P65 Interview} |
|  |  | I had a few massive panic attacks prior to my operation and had a couple of nurses literally had remorse and feelings for what I was going through and place a hand or 2 upon me and even rubbed my arm [P4 Survey Qual Response] | On the Monday or the Tuesday, I was nil by mouth cause I'm going in [to the operating theatre] at 5. Then at 5 they say, no, he's not going. But I was still nil by mouth, and it was after the orders for dinner for food. And they would have known about that through the day that that wasn't going to happen [P67 Interview] |
